# The impact of climate change on visitor destination selection: A case study of the Central Drakensberg Region in KwaZulu-Natal

**DOI:** 10.4102/jamba.v13i1.1161

**Published:** 2021-12-03

**Authors:** Nduduzo A. Ngxongo

**Affiliations:** 1 Department of Hospitality and Tourism, Faculty of Management Sciences, Durban University of Technology, Durban, South Africa

**Keywords:** climate change, tourism, visitor choice, destination, climate preferences, Central Drakensberg Region

## Abstract

Many variables influence visitors’ destination choices, for example, affordability, attractions, health and safety, and expectancies. Amongst the indirect influences in destination choice by tourists, climate change is perhaps the greatest factor because of its negative impact on the ecological landscape of tourist destinations. Using the Central Drakensberg Region (CDR) as a case study, this article seeks to investigate the influence of climate change on visitors’ destination choices. A quantitative descriptive survey was conducted on a sample of n347 participants who were selected using purposive and convenience sampling procedures under the auspices of non-probability. The statistical analyses were performed using the latest edition of the Statistical Package for the Social Sciences (Version 25.0). Based on the study’s results, climate change was found to be a key influence on tourists’ decision-making, behaviour and spending habits at tourist destination like the Central Drakensberg Area. The most significant climatic parameter for tourists was determined to be pleasant and warm temperatures, as well as the summer season. Furthermore, owing to the current climate change circumstances, visitors were found to be less likely to return or recommend future visits to the region. To that end, tourism officials in the CDR should swiftly implement adaptation measures to counter the effects, and explore alternatives such as soft tourism and non-climate-dependent activities to accommodate visitors year-around. Further research is recommended to establish the extent to which socio-demographic characteristics influence destination choice in the area, as well as to ascertain the current state of climate change impacts.

## Introduction

Tourism is one of the fastest growing and largest economic sectors in the world, serving individuals who travel from one place to another for recreation, whilst also generating revenue for the host destinations, communities, and/or countries (Sofronov 2018). Notwithstanding, Awojobi (2017); Dube and Nhamo (2018) argue that the sustainability and success of tourism has recently been threatened because of the phenomenon of climate change, which has and continues to be the greatest threat facing the tourism industry in the 21st century. The catastrophe of changing climatic conditions, global warming, and security problems, according to Gossling (2002), are causing increasing concern on a global scale. As a result of climate change, tourist concerns are growing, which is having a severe impact on the profitability of many destinations, as well as visitor decision-making, behavior, and spending patterns. Climate constitutes an important priority for many visitors when choosing which destination to visit. The attractiveness of a prospective destination is influenced by different variables (and/or people’s mental representations of the weather, that is, the psychological representation of weather) (Becken 2007; Bigano, Hamilton & Tol 2006; Dahiya & Batra 2016). Calm and warm weather is perhaps the most significant holiday expectation because they enable particular types of activities to be available at the destination and can also impact the accessibility of both the destination and activities depending on the time of the trip.

Climate change is projected to have a significant impact on the tourism industry’s future viability and sustainability (Dahiya et al. 2016). For example, in the Central Drakensberg Region (CDR), mountain tourism and ecotourism are a big tourist attraction, and are largely dependent on climate conditions, which are now expected to be highly unfavourable to activities like mountain hiking and other ecotourism attractions. Changes to tourism destinations as a result of climate change may have an adverse effect on tourists’ destination choices (Hamilton & Tol 2007; Scott & Lemieux 2010). Furthermore, Hamilton et al. (2007) does note that as a result of climate change globally, the attractiveness on domestic climates is likely to increase, thereby encouraging individuals in these areas to choose domestic getaways over international vacations. Furthermore, according to Nyaupane and Chhetri (2009), extreme weather occurrences as a result of climate change would have a detrimental influence on visitor safety, perhaps leading to trip cancellations or never returning to the same destination.

Climate is one of the macro-environmental variables over which tourism destinations have limited control, according to Hamilton (2003). However, Grant (2015) contends that there is a lack of awareness in the body of knowledge about how climate influences visitor travel decisions, perceptions, travel behaviour, and spending patterns, particularly in the context of South Africa, a topic that is one of the most relevant themes in climate change-tourism research. Temperatures are anticipated to rise in the uKhahlamba-Drakensberg Park, snow levels will decline rapidly, precipitation patterns will change, and the number of severe weather-related occurrences will likely increase as a result of changing climatic circumstances (Bentley & O’Connor 2018). As a result, potential tourists will have additional reasons to rethink about visiting the CDR, and visitors’ impressions of the region as a desirable destination will be affected.

Despite the fact that numerous studies on the impacts of climate change have been conducted in the past, the tourism literature review indicates that no research studies addressing the factors of climate change and tourist preferences of destinations have been conducted in South Africa to date. This study aimed to bridge that gap. The purpose of this article is to investigate the potential impact of climate change on destination choice, as well as how the impact may influence the choices and preferences of tourists visiting the region. The study also seeks to contribute to the tourism-climate change literature in the Central Drakensberg, which is currently not studied.

Considering that tourism is one of the region’s main economic drivers, tourism research is essential for KwaZulu-Natal and, in particular, the CDR. As a result, it is necessary to take into consideration the long-term impacts of climate change on tourism, which might have a negative impact on both supply and demand of tourism services in the region. Because the CDR is located in the mountains, ecotourism and nature tourism are prevalent, and tourism is a substantial economic engine in the region, making it susceptible to climate change.

This article argues that before embarking on a holiday, visitors conduct a thorough analysis of the climatic conditions of the tourist destination. According to Guliyeva (2018), visitors are increasingly becoming more informed as a consequence of their experiences, the worldwide web (internet), and word of mouth from friends and colleagues who have been to their desired destination. This study did not attempt to address all elements of a visitor’s preference for a destination, such as views, attitudes, reasons, or experiences. Its objective is to highlight climate and environmental variables as significant drivers of tourism demand. The study hypothesis is that climatic conditions in the CDR are a key issue for visitors, and that variations from tourists’ climate preferences and expectations would lead to negative perceptions of the travel experience, including negative perceptions of the destination’s value for money.

### Relationship between tourism and climate change

Following the discussion of climate change principles, it is important to explain the relationship between these two variables: tourism and climate change. According to Scott, Gossling and Freitas (2009), researchers and scholars began to pay attention to the relationship between tourism and climate change in the 1950s. This was mostly motivated by a need to learn more about how climate change affects tourism (Moreno & Amelung 2009). Indeed, there is a connection between tourism and climate change, as climate and tourist destinations are naturally integrated. More precisely, environment and weather are critical factors in the performance sustainability of tourism destinations, which is why tourism is particularly adaptive and vulnerable to climate change impacts. But, according to Gossling (2002), tourism also contributes to climate change by raising sea levels, emission of greenhouse gases, and other factors.

Because tourism is dependent on the climate, it is only natural that changes in the environment would have an effect on tourism. Rayamajhi (2012) investigated the relationship between tourism and climate change. He argued in the study that climate has a significant impact on the tourism and recreation sector, because it affects not only the natural resources that support tourism and recreation, but also the duration and nature of tourism and recreation seasons, tourist health, and even the quality of tourism experiences. Siddiqui and Imran (2018) further emphasised that because weather and environment are important factors in leisure demand, climate change may have an effect on tourism as a business.

Climate is an important commodity for tourism, especially for outdoor, nature-based, and weather-related tourism activities, according to Hamilton et al. (2007) and Grimm, Alcantara and Sampaio (2018). Changing climate and weather conditions in tourist attractions and tourist-producing countries may have a big impact on tourists’ comfort and travel plans. However, according to Martín (2005), the destination’s climate can be ‘sold’ as the main attraction. He discovered that warm temperatures in Spain in the summer were the biggest draw for outdoor activities, whilst ice-based activities were common in the winter. Broadly, the phenomenon of climate change will have negative consequences worldwide particularly for the tourism sector. Projections by Grimm et al. (2018) state that by 2050, the global tourism sector will be significantly impacted by climate change; hence, destinations and cities who use tourism as a major economic engine should take pre-emptive measures to adapt to that coming reality.

### Tourist destination choice literature

Many disciplines and application are interested in the effect of climate on visitor behaviour (Eugenio-Martin & Campos-Soria 2010). In general, there is general consensus amongst various disciplines that there is a strong correlation between climate and visitor behaviour (Becken 2007; Bigano et al. 2006; Dahiya et al. 2016; Guliyeva 2018; Lohman & Huebner 2013). In the context of this study, destination choice is described as a visitor’s choice of a specific destination from a collection of options (other destinations) (Hsu, Tsai & Wu 2009). Many people consider the process of selecting a destination to be a continuous decision-making process that begins with acknowledging the need to travel and ends with the final decision. Different factors motivate and affect certain decisions, with the weather and environment being one of the most common (De Freitas 2003; Karl, Reintinger & Schmude 2015). Essentially, visitors’ travel decisions are complex decisions in which the method of selecting various trip components is associated and changes over time. Several internal and external variables affect the visitors’ decision-making process (Kim & Prideaux 2005; Kozak 2002). Nonetheless, the influence of climate change on tourism destination choice in underdeveloped nations has received little consideration (Nicolau & Mas 2006).

Previous studies have concluded that the relevance and significance of climate and weather conditions in influencing tourists’ destination choice decision-making has been understudied and that empirical evidence is lacking (Bigano et al. 2006; Lise & Tol 2002; Lohmann & Kaim 1999; Tol & Walsh 2012). Climate and weather patterns according to Wilkins et al. (2017) are extremely important and can influence tourist destination selection, based on tourist demand for favourable temperatures and other factors. Furthermore, these variables may have a significant impact on spending patterns, visitor behaviour, activity habits, as well as the duration and nature of tourism seasons. Toubes, Araujo and Brea (2020) argue that for many years, climate has played a significant role in destination preference. Tourists’ satisfaction levels are greatly influenced by the environmental conditions that they encounter in their preferred destination for a variety of factors. For example, weather and climate can affect the behaviours of tourists or determine how pleasant an experience is. Therefore, these factors can impact tourists’ satisfaction levels which, in turn, influences their decision to revisit the destination (Grant 2015). Furthermore, one of the most significant reasons is the well-being of travellers, which can be jeopardised by extreme weather incidents and/or unfavourable climatic conditions (Siddiqui et al. 2018).

The climate is constantly changing and will continue to change in the future. Many academics now agree that the tourism industry will most likely change as the climate changes. According to Wilkins et al. (2017), there is still a great deal of uncertainty about how climate change could affect tourist travel decisions, attitudes, and spending habits. As a result, many tourist destinations are taking steps to mitigate the detrimental effects of climate change in order to attract tourists and maintain competitiveness. Enriquez and Bestard (2020) asserts that because of changing climatic conditions, the tourism industry is currently facing severe environmental and economic challenges. As a result, many visitors’ destination choices are greatly influenced (Jeuring 2017). Climate, without a doubt, is one of the most valuable natural assets for many tourist destinations, and plays an important role in tourists’ destination selections, spending patterns and behaviour. As a result of heavy reliance on favourable and enabling weather conditions, tourist destinations are vulnerable to any changes, especially the effects of climate change.

## Research context: Spatial setting

The study’s spatial context is the CDR, which is an area situated in the province of KwaZulu-Natal in the Republic of South Africa. It is regarded as the most attractive region of the Drakensberg Mountain (UKhahlamba Local Municipality Integrated Development Plan 2016). The magnificent CDR is a major part of the Drakensberg Mountain, which was declared as a World Heritage Site (WHS) by UNESCO in 2001 (Duval & Smith 2013). The tourism industry continues to be the most prosperous and noteworthy sector in the region, and has boosted the region, particularly the host communities, by generating employment opportunities, business opportunities, investment prospects, infrastructural development, and economic security (Mthembu 2011). However, despite the fact that global tourism growth, particularly in mountainous regions, has recently been hindered by a number of challenges, including climate change (Mukwada & Manatsa 2018), the region’s geographical location and outstanding landscape make it easy for it to become one of the country’s top tourism destinations.

## Research method

In general, there are three types of research methodology approaches: qualitative, quantitative, and mixed approach (ref). A quantitative research paradigm was used in this study. The quantitative method entails obtaining measurable data and applying statistical, mathematical, or computational tools to investigate phenomena in a systematic manner (Bryman & Bell 2015). In addition, an exploratory research technique was used in this study. According to Brown (2016), exploratory investigations are critical in determining what is happening in order to get new insights and investigate issues that have not previously been thoroughly investigated.

### Study population and sample size

The target population of this study was *N* = 450 respondents that consisted of two groups: stakeholders and experts. Stakeholders comprised of visitors at selected destinations, whereas experts comprised of Ezemvelo KwaZulu-Natal Wildlife (EKZNW) employees and the local municipality. The participating study areas were Didima Resort, Injisuthi Camp, Giants Castle, Cathedral Peak Hotel, Monks Cowl and Queen Elizabeth Park. To achieve the above, this study fused two different sampling techniques under the auspices of the non-probability sample method, namely purposive and convenience sampling. The sample size of the study is *n* = 350 respondents, broken down into the following different categories: *n* = 320 of the respondents were guests who visited the aforementioned destinations; *n* = 6 of the respondents were from the local municipality tourism department (the department had only 5 employees which means they all participated); and the last *n* = 24 respondents were from EKZNW.

### Data collection and analysis

To gather information, the researcher developed a questionnaire that included sections constructed from scratch as well as sections modified from Bigano et al. (2006); Tol et al. (2012); Guliyeva (2018). These instruments were relevant to this study because they addressed comparable variables of climate change impact on visitor choice and preferences. In terms of variables, geographic emphasis, type of questions, and format, the questionnaire was changed and updated accordingly. A pilot study was conducted in three distinct capacities to guarantee the findings’ reliability and consistency. The results of the pilot investigation were valuable in determining the scale of the empirical items of this investigation.

It was also discovered that several of the long and open-ended questions were somewhat difficult to comprehend, potentially resulting in inaccurate data that might undermine the findings’ validity. The study questionnaire’s guiding principle was drawn from a relevant literature review and tailored to the current research context through the use of study variables and a Likert-scale format. During the data collecting stage of the project, three (3) research assistants (Master’s degree candidates) were employed. To ensure quality data collection, the research assistants received training in interviewing, communication, climate change, and data collection.

From June 2018 to February 2019, data was collected at multiple sites through site visits. Given the tenets of ethical considerations (Shepard 2002), the study permit from the University was sought and received from the Faculty Research Committee and the Institutional Research Ethics Committee. Each questionnaire included a consent form and an information letter so that respondents could fully understand the study’s objectives and give their permission to participate. Beneficence (do no harm), regard for privacy, secrecy and confidentiality, and informed consent were amongst the main ethical clearance elements considered.

The information gathered was analysed using the most recent version of the Statistical Package for the Social Sciences (Version 25.0) at that time. The data were analysed using descriptive statistics and correlation matrix such as means and standard deviations. Internal accuracy was measured using Cronbach’s alpha coefficients. In terms of statistical significance, the Spearman correlation matrix was used to establish the practical relationship between the variables. Multiple regression analysis, exploratory component analysis, test of normality, Bartlett’s test for homogeneity of variances, and Levene’s test of equality were some of the key statistical analyses adopted in this study.

### Empirical findings and analysis

The questionnaire, which contained 89 Likert scale typed statements, was sent to 320 conveniently selected respondents (visitors) from the selected tourism destinations. With 317 individuals completing the questionnaire, the response rate was about 99%. The Cronbach’s Alpha test was utilised as a measurement item for internal consistency and construction reliability. A dependability coefficient of 0.60 or greater for a newly constructed structure is deemed ‘acceptable’ (Bryman et al. 2015). The reliability scores for each component were all greater than the recommended Cronbach’s alpha rating. Precisely, travel behaviour received a 0.66 score, visitor spending patterns received a 0.63 score, destination choice received a 0.82 score, and climatic preferences received a 0.79 score.

### Demographic characteristics of respondents

Table 1 reveals that the response rate of male respondents (54.9%) was slightly higher than that of female respondents (45.1%). The male-to-female ratio is approximately 1:1 (54.9%: 45.1%) (*p* = 0.082). The majority of participants (56.5%) were between the ages of 18 and 30, with the lowest percentage (51 to 60) falling between those ages. (1.6%). In terms of nationality, the great majority of the respondents (66.9%) were South Africans, whilst the other 105 (33.1%) were international visitors. According to the educational credentials, the majority of respondents (> 50%) held a post-school certification (national certificate = 2.8%, national diploma = 29.7%, and postgraduate degree = 20.2%). Lastly, the survey found that the majority of respondents (121; 38.2%) had only visited once, whilst 100 (31.5%) had visited twice. It is also worth mentioning that 22 (6.9%) of the 317 survey respondents indicated that they were regular visitors, with 25 (7.9%) indicating that they visited the CDR more than five times.

**TABLE 1:** Analysis of demographic data of the respondents.

Variable	Frequency	Per cent
**Age**		
18–30	179	56.5
31–40	109	34.4
41–50	6	1.9
51–60	5	1.6
61–70	9	2.8
Cannot disclose	9	2.8
Total	317	100.0
**Education**		
No education	40	12.6
Grade 12	95	30.0
National certificate	9	2.8
National diploma	94	29.7
Postgraduate degree	64	20.2
Cannot disclose	15	4.7
Total	317	100.0
**Nationality**		
Local	212	96.5
International	105	33.5
Total	317	100.0
**Purpose of visit**		
Holiday	99	31.2
Family and friends	55	17.4
Flora and fauna	115	36.3
Business	14	4.4
Education	24	7.6
Sport	10	3.2
Total	317	100.0
**Visit frequency**		
Once	121	38.2
Twice	100	31.5
Thrice	49	15.5
More than five times	25	7.9
Regular visitor	22	6.9
Total	317	100.0

### Climate preferences and travel destinations

In order to determine climate preferences, visitors were given a wide range of climate parameter variables to choose from, including wind, humidity, temperature, rain, snow, frost, fog, and air pressure. The most significant climate factors for visitors when choosing a place were consistently evaluated as temperature, wind, rainfall, and snow. Temperature, on the other hand, rated far higher than the other factors, and this would be the focus of the investigation. According to Rossello and Santana (2014), temperature is the most significant climatic variable for tourists when choosing a destination. As a result, it was considered appropriate to probe more deeply into the research participants’ wide variety of temperature choices.

According to the findings, the optimal temperature for sightseeing visitors was around 26°C and 29°C, with another 29% preferring temperatures of 30 degrees or above. A clear sky with no mist or fog, as well as great visibility, were highlighted by visitors as indicative of ideal weather for sightseeing and outdoor activities. The visitors who stated that flora and wildlife as their major reason for visiting, preferred temperatures ranging from 26°C to 30°C. A warmer temperature was preferred for hiking routes and camping, suggesting a daily maximum temperature of approximately 18–24°C. According to the data, visitors appreciate weather conditions with a pleasantly mild wind, but strong winds were opposed by many visitors. Summer season, according to the majority of respondents, (169/60%), was identified as the most ideal time to visit the CDR in terms of climate period. The respondents cited long summer days, warm temperatures, breathtaking species of flora and wildlife, and other characteristics as reasons for summer visits.

However, the lushness and greenery of the peak, as well as the enhanced opportunities for sight-seeing, are the major reasons for visiting CDR in summer. Despite being aware of the heightened risk of flooding and potentially catastrophic weather conditions associated with summer, the respondents said that the weather was a key incentive for them.

Furthermore, a significant number of respondents (69; 30%) indicated that winter is their preferred season for visiting CDR. According to these responses, the primary reasons for visiting the region are hiking, camping, and the low likelihood of rain. The winter season in the Great Drakensberg is characterised by cold temperatures, prolonged drought, and a thick blanket of snow. The weather is believed to be perfect for hiking trails at this time of year. Surprisingly, rather than the environment and weather conditions, a tiny proportion of respondents (32; 10%) identified affordability, travel to new destinations, and special offers as the major reasons for visiting CDR. This implies that weather and the environment are not, at all times, major factors for some of these respondents when choosing a vacation destination, nor does it influence their spending patterns.

The visitors were also asked if they expected to visit the CDR again in the near future, in order to establish whether the CDR’s climate had an impact on their future travel plans. Unsurprisingly, the weather had a significant impact on the respondents, who claimed that a repeat visit was uncertain (64%). These participants indicated an unlikely or undetermined intention to re-visit the destination. When asked about the best times to visit the CDR, many participants, particularly those from outside of South Africa, indicated the mid-year and end-of-year periods. Such periods correlate to the winter in the South, which is often dry and warm with snowfall in hilly areas such as the CDR. The end-of-year seasons are characterised by sunny and pleasant weather, as well as torrential downpours, which provides the greatest landscape for travellers. Climate conditions may affect tourism demand directly, influencing destination choice and the possibility of repeat visits, as well as indirectly, by influencing the quality of the experience, formulating negative perceptions, and uncertainty about a destination’s attractiveness (Semenza & Kristie 2019).

Altogether, the findings show that a pleasant environment with moderate temperatures is an important travel incentive, influencing not just travel behaviour but also destination choice, visitor satisfaction, and travel duration. As a result, increasing our understanding of visitor climate choices is both a thought-provoking and fundamental academic topic that must be critically engaged if accurate climate change implications of visitor’s choice of destination are to be better understood.

### Climate change and visitor travel decisions

When asked about their pre-travel behaviour, a significant majority (90.7%) of respondents stated that they looked at climatic conditions in prospective tourist destinations before deciding on a vacation. When asked if they will choose a holiday destination based on whether or not it is actively involved in climate change adaptation, a significant majority (84.5%) stated that they will primarily choose prospective holiday destinations based on the level of climate change adaptation commitment. Furthermore, when asked about the specific weather conditions that would induce them to cancel their trip to the Drakensberg, the majority (75.7%) consistently mentioned heavy rain and strong winds as the climatic factors that would cause them to cancel their excursion, followed by high temperatures (48.9%), all of which support the assertion that climatic conditions have a significant impact on tourism in the Drakensberg.

The preponderance of visitors (65.3%) choose the CDR as a tourism destination because of the pleasant weather. Given the significance of climate to visitors, it only seemed reasonable to inquire if they evaluated the climatic conditions of their destination before making a reservation. Surprisingly, a huge majority (93.0%) indicated that they checked the weather forecast for their holiday destinations. These findings corroborate with Guliyeva’s (2018) conclusions that climate change has a major influence on tourism destination choices and that inclement weather is more likely to deter tourists from visiting a prospective destination.

### Visitor spending patterns and behaviour

Weather patterns are expected to have a significant impact on visitor spending and behaviour, as well as the economy of tourism-dependent regions like the CDR. The impact of climate change on visitor travel expenditures and behaviour was determined using a Chi-Square test using *p*-values. Because the prospective destinations are intensely interested and engaged in climate change initiatives, 72.2% of respondents indicated that they would prefer such a destination. Furthermore, 93.4% agreed with the statement ‘I am glad to invest my money in an environmentally sustainable destination’. This implies that the majority of respondents are inclined to travel to destinations that are ecologically friendly and sustainable.

Despite this, just 51.2% indicated that they would be prepared to spend increased rates to help with climate change adaptation expenditures, whilst 38.3% said they would not. This clearly illustrates that not all visitors will be able to afford increased fees in order to aid in the fight against climate change. However, the overwhelming majority of respondents (96.9%) agreed that climate change had an impact on tourist spending patterns and behaviours. In addition, the vast majority of respondents (97.8%) indicated that they would postpone their vacation if the weather conditions were unpleasant owing to the region’s unfavourable climate. These findings are confirmed by the fact that 89.5% stated they would not travel to the destination if the weather conditions were unpleasant. Overall, the findings suggest that climate change is indeed a significant factor in visitor choices and spending habits, and that environmentally friendly locations are more likely to attain better profit margins than environmentally unfriendly destinations.

### Descriptive statistics and correlation matrix

Table 2 presents the descriptive statistics and correlation matrix for the study following questionnaire items: CW-Climate and weather conditions, PD-Popularity as a renowned destination, QP-Quality and the price of accommodation, LD-Cleanliness of destination, SS-Safety and security, EA-Easy accessibility of a destination, C0-Comprehensive online information/content, QS-Quality of the area’s natural setting, CH-Cultural and historical attractions, AS-Availability of special and discount offers, PL-The physical images and presentation of the location, CC-climate change combat initiatives, EF-environmentally friendly, AC-accommodation prices, VS-Visitor’s spending pattern, VC-vacation conditions, DU-destination unfavourable.

**TABLE 2:** Descriptive statistics and correlation matrix.

Variable items	*N*	Mean	Standard deviation	CW	PD	QP	LD	SS	EA	CO	QS	CH	AS	PL	CC	EF	AC	VS	VC	DU
CW	317	3.02	1.37	1.00	0.643	0.579	0.554	0.506	0.643	0.579	0.579	0.554	0.554	0.506	0.643	0.579	0.579	0.579	0.554	0.554
PD	317	3.22	1.11	0.643	1.000	0.865	0.660	0.721	1.00	0.865	0.864	0.660	0.660	0.721	1.000	0.865	0.862	0.660	0.660	0.721
QP	317	3.34	1.33	0.579	1.000	1.00	0.506	0.643	0.721	0.579	0.621	0.554	0.772	0.506	1.000	0.643	0.721	0.579	0.621	0.579
LD	317	3.22	1.07	0.554	0.867	0.506	1.000	0.865	0.621	0.554	0.773	0.554	0.771	0.865	0.860	0.863	0.721	0.721	0.686	0.686
SS	317	3.08	1.13	0.506	0.865	0.643	0.621	1.00	0.721	1.00	0.865	0.865	0.660	0.660	0.721	1.000	0.865	0.864	0.660	0.660
EA	317	3.22	1.31	0.643	0.660	0.721	0.554	0.660	1.00	0.643	0.579	0.554	0.506	0.643	0.579	0.579	0.554	0.554	0.506	0.643
CO	317	3.35	1.33	0.579	0.660	0.579	0.773	0.721	0.554	1.000	0.506	0.643	0.721	0.579	0.621	0.554	0.773	0.506	0.643	0.721
QS	317	3.34	1.06	0.579	0.721	0.621	0.554	0.721	0.506	0.554	1.000	0.554	0.773	0.554	0.773	0.865	0.863	0.554	0.773	0.554
CH	317	3.24	1.07	0.554	0.721	0.554	0.773	0.660	0.643	0.506	0.506	1.000	0.506	0.643	0.721	0.579	0.621	0.554	0.773	0.506
AS	317	3.24	1.13	0.554	1.000	0.773	0.863	0.579	0.579	0.643	0.643	0.554	1.00	0.867	0.865	0.660	0.660	0.721	0.865	0.865
PL	317	3.08	1.31	0.506	1.000	0.506	0.864	0.621	0.579	0.579	0.721	0.773	0.554	1.000	0.863	0.621	0.554	0.773	0.554	0.773
CC	317	3.22	1.33	0.643	0.865	1.000	0.865	0.554	0.554	0.579	0.579	0.554	0.506	0.643	1.000	0.506	0.643	0.721	0.579	0.621
EF	317	3.32	1.33	0.579	0.865	0.643	0.686	0.773	0.554	0.554	0.621	0.773	0.621	0.554	0.773	1.000	0.864	0.861	0.660	0.660
AC	317	3.31	1.33	0.579	0.865	0.721	0.686	0.579	0.506	0.579	0.579	0.554	0.554	0.506	0.643	0.579	1.000	0.506	0.643	0.506
VS	317	3.32	1.06	0.554	0.865	0.579	0.686	0.579	0.506	0.643	0.721	0.579	0.621	0.554	0.773	0.506	0.643	1.000	0.773	0.554
VC	317	3.24	1.07	0.554	0.660	0.621	0.621	0.865	0.643	0.579	0.554	0.506	0.643	0.579	0.579	0.554	0.554	0.506	1.000	0.506
DU	317	3.24	1.06	0.554	0.660	0.579	0.621	0.864	0.660	0.660	0.721	1.000	0.865	0.865	0.660	0.660	0.721	0.865	0.660	1.000

CW, climate and weather conditions; PD, popularity as a renowned destination; QP, quality and the price of accommodation; LD, cleanliness of destination; SS, safety and security; EA, easy accessibility of a destination, CO, comprehensive online information/content; QS, quality of the area’s natural setting; CH, cultural and historical attractions, AS, availability of special and discount offers; PL, the physical images and presentation of the location; CC, climate change combat initiatives; EF, environmentally friendly; AC, accommodation prices; VS, visitor’s spending pattern; VC, vacation conditions, DU, destination unfavourable.

Table 2 also depicts the inter-construct Spearman correlation matrix for the research variables, as well as a summary of descriptive statistics (i.e. mean and standard deviation). It shows a mean of 3.02 for climate and weather conditions suggesting that, on average, the participants rated the climate and weather conditions item the least, whilst comprehensive online information/content item was rated the highest by the participants. The correlation between the variables is also shown in Table 1. As a measure of the strength and direction of correlation, the Spearman correlation matrix was utilised. Table 1 also demonstrates the following significant positive correlations between the following items: PD, QP, LD, SS, EA, CO, QS, CH, AS, PL, CC, EF, AC, VS, VC, DU. However, there was no significant correlation with CW.

Significant panel regression results were reported for the two research variables, namely Climate Preferences and Destination Choice, and Visitor Behaviour and Spending Pattern. The results adjusted for heteroskedasticity will now be presented. The panel regression results are shown in Table 3, and it shows that there is a significant positive relationship between visitor behaviour and spending pattern, and tourism destination choice. The panel regression results also show that there is a significant positive relationship between climate preferences and tourism destination choice.

**TABLE 3:** Panel regression results.

Variable	Regression coefficient	Standard error	*T*-value adjusted for heteroskedasticity	Pr > |*t*|
Climate preferences and destination choice	−0.059	0.033	−1.761*	0.040
Visitor behaviour and spending pattern	0.534	1.022	4.134**	0.000

* , Significant at the 5% level;

** , Significant at the 1% level.

Table 4 shows the results of a regression analysis performed to verify the hypotheses of this study, on the relationship between Climate Preferences and Destination Choice, and Visitor Behaviour and Spending Pattern on Tourism destination choice, and it was found that climate Preferences and destination choice positively affected tourism destination choice (β = 0.995, *t* = 0.996, *p* = 0.001; adjusted *R*² = 0.994, *F* = 58.397). Table 4 also shows that Visitor Behaviour and Spending Pattern positively affected Tourism destination choice (β = 0.626, *t* = 0.038, *p* = 0.001; adjusted *R*² = 1.000, *F* = 16.444).

**TABLE 4:** Linear regression analysis results.

Variable	No. of items	*B*	β	SE	*t*	*p*	Adjusted *R*^2^	*F*
Climate preferences and destination choice	11	0.997	0.995	0.857	0.996	0.001	0.994	58.397
Visitor behaviour and spending pattern	6	0.626	0.038	0.633	1.000	0.001	1.000	16.444

Table 5 presents the results of the exploratory factor analysis (EFA) used in this study to determine the relationship between the study variables. It found two distinct factors that explained 80.33% and 10.60% variance, respectively, with factor loading ranging from 0.884 to 0.968 and reliability values of 0.966 and 0.961.

**TABLE 5:** Exploratory factor analysis result.

Factors	Items	Factor loading	Cronbach’s alpha	Variance explained (%)
Climate preferences and destination choice	11	0.884	0.966	80.332
Visitor behaviour and spending pattern	6	0.968	0.961	10.597

The results of the Pearson correlation tests also confirmed relationships between Climate Preferences and Destination Choice and Tourism destination choice on one hand, and Visitor Behaviour and Tourism destination choice on the other hand. These results confirm that there are relationships between all the variables proposed by the initial hypotheses of this study (see Table 6).

**TABLE 6:** Correlation between variables.

Variable	Visitor destination choice	Climate destination choice
Visitor destination choice	1.000	0.997*
Climate destination choice	0.997*	1.000

* , Correlation is significant at the 0.05 level (2-tailed).

The normality tests for the total score of the two constructs of this study were conducted using Kolmogorov-Smirnov and Shapiro-Wilk in order for this research to accomplish its objectives. The results of normality tests are reported in Table 7 for Climate Preferences and Destination Choice and Tourism Destination Choice on one hand, and Visitor Behaviour and Tourism destination choice on the other hand. It shows that data were normally distributed (*p* < 0.05), and that both Climate Preferences and Destination Choice, and Visitor Behaviour significantly affects tourism destination choice (see Table 6).

**TABLE 7:** Tests of normality.

Test	Statistic	Degrees of freedom	Significance
Kolmogorov–Smirnov†	0.450	32	0.001
Shapiro–Wilk	0.565	32	0.001

† , Lilliefors Significance Correction.

For all of the variables in this study, the Bartlett’s test was employed to verify that the variances were equal. The results of the Bartlett’s test for homogeneity of variances are summarised in Table 8, and the *p*-value indicates that homogeneity of variances has occurred (*p*-value at 0.001 which is less than 0.05). This is supported by Levene’s test of equality (Table 9), which shows that the obtained similarities between the variances in the samples for pre- and post-data occurred at *p*-value 0.000, which is less than the statistically significant threshold of 0.05.

**TABLE 8:** Bartlett’s test for homogeneity of variances.

Variables	Means of transformed data	Standard deviations of transformed data	*P* value
Visitor destination choice	128.589	9.99	0.001
Climate destination choice	88.96	8.96	0.001

**TABLE 9:** Levene’s test of equality.

Levene – statistic (based on means)	Significance
128.589	0.001

## Discussion of findings

The study’s geographic location has significant implications. It is a heliocentric destination that primarily attracts visitors because of its environment and it is also located in an area that is most vulnerable to expected climate change. As a result, the potential interactions between climate and tourist activities may provide some valuable information for visitor decision-making.

Simply explained, tourists are more inclined to choose a destination, revisit, recommend it to other potential visitors, spend a significant amount of money, and behave ethically when the environment and climatic conditions in the region are favourable. The consequences of climate change on any tourist destination in the future may have a negative impact on visitors’ destination choices (Becken & Wilson 2013). As a result, changing climatic conditions in the CDR are already having an impact on visitor destination choice and will continue to do so. The changes in destination climatic patterns also prompt visitors to reconsider their plans and migrate to destinations with better climate conditions. Furthermore, tourists must feel at ease throughout their outings. As a result, unpleasant and inclement weather, such as excessively low temperatures or unexpected weather changes, may somehow have detrimental impacts. Adverse climate conditions are correlated to a tourism deficit, according to Toubes et al. (2020). This indicates that, as expected, there is a cumulative movement of visitors from colder to warmer destinations.

Because of the deteriorating environment and other unfavourable conditions, the majority of respondents were unwilling to revisit the CDR. The findings contradict those of Lohmann and Huebner (2013), who concluded that tourists in La Martinique were undeterred by the climate and overwhelmingly indicated that they would return, with over 80% of respondents expressing a strong wish to return to the island. Similarly, the data supports the assertion that pleasant temperatures are the most significant climatic parameter for tourism, and that climate has impacted and will continue to influence many elements of tourists’ decision-making processes. Similarly, the majority of visitors indicated that they are pleased to spend their money in environmental-friendly destinations and regions. As a result, destinations that are ignorant about the environment are more likely to incur considerable losses. Visitors can now pay higher accommodation rates to assist authorities in addressing the financial expenses associated with climate change adaptations. These visitors’ contributions are likely to go towards the financial coffers that will be used to compensate for the losses caused by climate change. Climate change has had a major influence on visitor spending patterns and behaviours, according to the study’s findings. Climate change has harmed the tourism sector in the area, thus lowering its financial contribution and endangering the long-term viability of local employment prospects.

The impact of climate change on tourist destination choices may have disastrous economic repercussions for both the CDR and the tourism sector in KZN, because visitor spending habits and travel patterns are generally severely influenced when weather conditions are poor. The respondents’ destination preference is strongly correlated with a destination’s physical characteristics, in contrast to socio-demographic and travel motivation factors (i.e. landscape, atmosphere, soil, natural vegetation, and plant and animal life). The findings are interesting because Jang and Cai (2002) underlined the relevance of travel motivation variables on destination in a previous study on the same topic. Socio-demographic variables, according to Bigano et al. (2006), have a significant influence on destination choices.

It is apparent that an unstable climate will indeed deter visitors for a number of reasons, thus indicating that, in addition to the destination, visitors evaluate the climate in various aspects of their decision-making process. Scholars from all across the world have corroborated this argument. According to Becken (2010), as individuals become more conscious that climate change is a significant factor influencing both visitor travel decisions and tourism activities, they are becoming more cognisant of the critical role that climate has in the tourism business. A safe environment and ideal climate conditions, according to Mishev and Mochurova (2010), seem to be critical to visitor satisfaction and is a primary factor for visitors when choosing a destination. Warmer and more pleasant climate boosts tourism expenditure, particularly in the summer, whilst the impacts were more mixed in the winter (Wilkins et al. 2017). From a long-term and cost-effective standpoint, it may be argued that destinations with more resilience capabilities are more likely to expand their market share and profitability as more visitors choose climate-adaptive and environmentally sustainable destinations.

### Study limitations

This study had the following limitations:

- The lack or non-existence of a tourism theoretical framework meant that the study had to opt for a conceptual framework in tourism climatology, which is of course not a full-blown framework.
- It is moreover essential to comprehend that the field of tourism climatology is yet to gain significant academic attention, thus resulting in limited academic literature;
- The study’s spatial settings are only limited to the CDR. Considering other regions may have contributed new and important information to the study; and
- Limited outcomes in quantitative research. The respondents had limited options of responses based on the selection made by the researcher. As such, generalisation of the results is limited.

### Study implications

Tourism businesses in the CDR should consider examining the correlation between climate change and tourist decisions or choices, and implement strategies, policies, and processes that will help to generate reciprocal benefits. The findings of this study may be utilised as a decision-making mechanism and therefore can offer insights into the relationship between climate change and tourist destination choice. The findings also inform local, regional, and national policymakers on the importance of climate change mitigation and adaptation in strengthening tourism destination sustainability whilst also protecting biodiversity. The study will help tourism officials better understand some of the main elements and indicators that impact tourists’ destination choices.

### Suggestion for future study

Further research is recommended into macro (external) and micro (internal) environment variables that influence visitors’ decision-making and travel decisions in the tourism industry. The research further recommends examining the extent to which socio-demographic factors such as education, marital status, gender, income, and age impact and/or decide tourists’ travel decisions and behaviours when choosing a destination.

## Conclusion

This study produced a number of noteworthy findings. Climate was highlighted as a major tourism variable as well as an essential destination attribute in visitors’ decision-making processes. The most significant tourism resource is the climate; for example, in natural destinations such as the CDR, it acts as a facilitator, attracting tourists and facilitating tourism activities. This study provides tourism destinations with valuable information that can be used to improve their business strategy. Climate change factors such as precipitation, wind, humidity, temperatures, ecosystems, and animals all have a favourable impact on tourist destination choices, spending habits, and travel behaviour. Although service quality, word-of-mouth, pricing, and reputation are all important factors, the extent to which weather and environment may impact tourists’ choice is an important element that should, at all times, be considered. Given the severity of potential climate change implications, this article recommends that the CDR should engage on implementing a set of adaptation and mitigation measures as soon as possible to mitigate climate change impacts and create an environment that meets tourist expectations. Furthermore, the study recommends that the CDR consider an alternative plan that promotes shoulder seasons or other types of tourism, such as soft tourism and non-climate-dependent activities like cultural, business, adventurous, and sports activities, so that tourists are catered for during peak and off-peak seasons.
